# The Dynamics of Becoming a Mother during Pregnancy and After Childbirth

**DOI:** 10.3390/ijerph17010057

**Published:** 2019-12-19

**Authors:** Anna Zdolska-Wawrzkiewicz, Mariola Bidzan, Magdalena Chrzan-Dętkoś, Daria Pizuńska

**Affiliations:** Institute of Psychology, Faculty of Social Science, Univerity of Gdansk, Bazynskiego 4, 80-952 Gdansk, Poland; mariola.bidzan@ug.edu.pl (M.B.); psymcd@ug.edu.pl (M.C.-D.); daria.pizunska@ug.edu.pl (D.P.)

**Keywords:** self-perception, attachment, parenting, pregnancy, transition to motherhood

## Abstract

Background: The aim of this study was to explore the relationship between one’s maternal attachment style and one’s self-image as a mother, image of one’s mother as a mother, and bond with the child over a period of several months. Methods: A total of 86 women took part in the study The Adjective Check List (ACL), Postpartum Bonding Questionnaire (PBQ), Maternal–Fetal Attachment Scale (MFAS), and a modified version of the Experiences in Close Relationships (ECR) were used. Two measurements were used: during pregnancy and about six months after the birth of the child. Results: In terms of their self-image as mothers, the women had higher results the first time they took the questionnaire, regardless of their attachment style. An interaction effect was found between attachment style and the ‘need for changes’ scale. The image of one’s mother as a mother depended on the level of avoidance in attachment, regardless of the measurement. An interaction effect was found between attachment style and the scale of ‘personal adaptation’. There was a correlation between the bond with the child during pregnancy, the bond following birth, and the style of maternal attachment; the main predictor of the bond with the child after birth is the bond with the child during the pregnancy. Conclusion: Those who provide care for pregnant women and new mothers should be aware of the complex psychological processes in the transition to motherhood, have knowledge about perinatal mental health, and when necessary, refer women to specialists such as support groups for new mothers, trained midwives, psychologists, psychotherapists, or psychiatrists.

## 1. Introduction

The formation of one’s identity as a mother begins in childhood with a female identifying with her parents. The decision to have a child and the pregnancy itself, intensify the formation of a maternal identity [1]. This process is particularly intense in the second trimester of pregnancy [2]. Subsequently, by about four months after birth, one’s self-image as a mother becomes ‘autonomous’ and separates from the image of one’s mother as a mother [1]. The process of identification with the role of mother is related to one’s maternal attachment style [3,4,5]. One’s own experiences with attachment figures, and if a couple plans and experiences pregnancy are also important factors that influence the transition to motherhood [6,7,8,9,10]. What is interesting is that although almost half of all pregnancies in the United States are unplanned [11], most families are able to adapt to these unexpected circumstances [12]. However, the maternal–fetal attachment level in unplanned pregnancies is weaker than their planned counterparts [13] which can influence further maternal–child bonding and child outcomes. Acknowledging that many factors influence maternal–child bonding such as planned vs. unplanned pregnancy, parental stress, occurrence of depressive symptoms, and so on, due to limitations related to the volume of the text, we would like to focus on the maternal attachment style and one’s self-image as a mother, image of one’s mother as a mother, and bond with the child over a period of several months.

Our previous research showed that maternal attachment style is related to one’s image of one’s mother as a mother [14]. In pregnancy, this affects a woman’s need for autonomy and parental responsibility (i.e., women with a less secure attachment style got significantly higher scores on the ‘need for autonomy’ scale in the Adjective Check List (ACL) questionnaire). After the birth of a child, maternal attachment style influences both one’s self-image as a mother and the image of one’s mother as a mother. Women with a secure attachment style described their mothers using many more positive and fewer negative adjectives: the image of their mothers was fuller—the mothers were described as both caring and capable of taking care of themselves. In contrast, women with a less secure attachment style perceived their mothers more negatively—as less confident and less caring [3,14].

In this paper, we focus on the dynamics of women’s self-image as dependent on their maternal attachment style. We also explore the dynamics of a mother-child bond preceding and following childbirth.

We consider the following three questions:

In women with different maternal attachment styles, does self-image as a mother change following childbirth?

In women with different maternal attachment styles, does the image of one’s mother as a mother change following childbirth?

In women with different maternal attachment styles, does the bond with the child change following childbirth?

## 2. Methods

The study involved two stages—the first stage was attended by 206 participants (the data were collected in clinics and hospitals) and the second stage by 86 participants. The females first completed the questionnaire in several antenatal clinics (93 females), or in hospitals (72 females) in Gdansk, Poland. This research obtained consent from the Ethical Commission of the Institute of Psychology of the University of Gdansk. In the second stage, while contact was made with all the surveyed women, a significant number did not respond to the invitation, could not participate in the study, or did not complete the questionnaires. In this paper, we describe the 86 women who took part in both stages of the study: during pregnancy and about half a year following childbirth (the date of contact was calculated based on the expected date of birth). The women were between 23 and 41 years old (mean (M) = 30.55; standard deviation (SD) = 3.775). Most were married (*n* = 65), had higher education (*n* = 72), and lived in a large city (*n* = 47). Most were first-time mothers (*n* = 74), gave birth by the estimated date of birth (*n* = 77), and had one child (*n* = 81). Most had a vaginal birth (*n* = 32 naturally and *n* = 17 induced).

Comparisons were made between those women who only completed the first data entry and those who completed both data sets. In the majority of the tested variables, there were not any significant differences. Compared groups of women differed in number of children (t = −3.553; *p* < 0.001), ‘critical parent’ in self-image as a mother (t = 2.059; *p* = 0.041), and also in the image of one’s mother as a mother: ‘need for achievement’ (t = 2.164; *p* = 0.032), ‘need for domination’ (t = 2.254; *p* = 0.025), ‘need for understanding oneself and others’ (t = 2.073; *p* = 0.039) as well as ‘personal adaptation’ (t = 2.171; *p* = 0.031).

During both stages of the study we used the following:

The Adjective Check List (ACL) was developed by Gough and Heilbrun [15] with a Polish adaptation by Martowska [16]. Participants completed this questionnaire twice, choosing from a list of 300 adjectives: first, those best characterizing them as a mother (′me as a mother′), and subsequently those best characterizing their mothers as a mother (′my mother as a mother′). Cronbach’s alpha ranged from 0.43 (on the ‘need for changes’ scale) up to 0.94 (on the ‘number of positive adjectives’ scale). We chose the ACL due to its complexity and the possibility of a multi-dimensioned approach to personality.

The Edinburgh Postnatal Depression Scale (EPDS), with a Polish adaptation by Kossakowska [17], is a set of 10 screening questions. It is likely that parents who score 12 or more points are suffering from perinatal depression [18].

We also used our own socio-demographic survey aimed at collecting sociodemographic data.

When considering issues regarding pregnancy and childbirth, it is important to consider perinatal depression. This disease affects about 10–15% of women [19,20,21,22,23,24]. Therefore, this issue was taken into account in our research and depression was a controlled variable in this study.

In the first stage we also used:

The Experiences in Close Relationships (ECR) questionnaire [25] with a Polish adaptation by Marchwicki [17]. This questionnaire measures two dimensions of attachment to a partner in a romantic relationship: avoidance and anxiety. Depending on the results obtained in these dimensions, the subjects are classified into four attachment styles: secure, preoccupied, avoidant, and anxious. The Polish version of the questionnaire was adapted to retrospectively measure maternal style of attachment. As a result of this, it classifies participants into two styles/dimensions of attachment: anxious-ambivalent and avoidant. Subjects were divided into groups according to their results in particular dimensions. Four groups were formed:

Group 1: high ambivalence/anxiety and high avoidance. *n* = 24

Group 2: low ambivalence/anxiety and low avoidance. *n* = 28

Group 3: high ambivalence/anxiety and low avoidance. *n* = 17

Group 4: low ambivalence/anxiety and high avoidance. *n* = 17

The reliability of scales was calculated with the Cronbach method (*n* = 302, Polish sample). Cronbach’s alpha was 0.89 and 0.68 for scales measuring avoidance and anxiety, respectively. The Polish adaptation consists of 19 questions with answers given on a seven-point Likert scale ranging from 1—’I definitely do not agree’ to 7—’I definitely agree’ [26].

Marchwicki obtained the consent of the authors of the original ECR version for the adaptation of the tool. The basis for agreement was a common theoretical background.

The Maternal–Fetal Attachment Scale (MFAS) was developed by Cranley [27] with a Polish adaptation by Bielawska-Batorowicz [28]. This questionnaire measures the bond with the child during pregnancy. Attachment to the child is measured in five dimensions: assuming a parental role, treating the child as an individual entity, establishing interactions, ascribing properties, and subordinating oneself to the child. Women marked their own answers on a four-point Likert scale. The higher the women scored on the 21 questions, the greater their attachment to the child. The reliability of particular scales in the Polish version is (Cronbach’s alpha) 0.66 for assuming a parental role, 0.58 for treating the child as an individual entity, 0.52 for establishing interaction, 0.61 for ascribing properties, and 0.64 for subordinating oneself to the child. For the entire test, Cronbach’s alpha was 0.81.

In the second stage we used:

The Postpartum Bonding Questionnaire (PBQ) [29] is a questionnaire designed to detect disorders of the mother–infant relationship. It includes four scales:

(a) impaired bonding;
(b) rejection and anger;
(c) infant-focused anxiety;
(d) incipient abuse.

All statistical analyses were performed using SPSS 25.0 (IBM, Chicago, IL, USA) software packages. In order to answer the first and second questions the authors conducted a mixed model ANCOVA – Analysis of Covariance (2 × 4) where the within-subjects variable was dichotomous before and after childbirth and the between-subjects variable was the attachment style of the examined women. Four factor levels of the attachment style were obtained by setting the level on anxious-ambivalent and avoidant scales in the Experiences in Close Relationships questionnaire. In the third question authors used a hierarchical regression analysis where the dependent variable was the bond with the newborn child (measured by the PBQ test). The predictor introduced in the first block was the connection with the child during pregnancy (measured by the MFAS test) and the predictors introduced in the second block were: anxiety-ambivalent style and avoidance style. The *t*-test was utilized to compare the differences between women who only completed the first data entry and those who completed both data sets. 

## 3. Results

We present the results in relation to each of the research questions.

Question 1: In women with different maternal attachment styles, does self-image as a mother change following childbirth?

In order to answer this research question, a mixed model ANCOVA was conducted, where the within-subjects variable was dichotomous before and after childbirth and the between-subjects variable was the attachment style of the examined women (four factor levels: high ambivalence/high avoidance, high ambivalence/low avoidance, low ambivalence/high avoidance, and low ambivalence/low avoidance). The continuous dependent variable was one’s self-image as a mother measured by the ACL questionnaire (each of the 37 sub-scales was analyzed separately), while the covariate was the level of depression measured after childbirth (ESDP).

The differences between the measurements were significant in terms of the following sub-scales: ‘complete list of adjectives’, ‘negative adjectives’, ‘typicality’, ‘the need for affiliation’, ‘leadership’, and ‘critical parent’ (see Table 1). In each of these sub-scales, participants’ scores were higher before childbirth, and after childbirth the level of these variables decreased significantly.

The effect of the main factor ‘attachment style’ turned out to be insignificant on all tested variables. The results showed a significant interaction effect between the factors of ‘measurement’ and ‘attachment style’ on the ‘need for changes’ subscale (*F* = 3.425; *p* = 0.021; *η^2^* = 0.113).

The analysis of simple effects showed that women with high levels of anxiety and avoidance had a significantly stronger ‘need for changes’ before childbirth than after childbirth (*F* = 4.008; *p* = 0.049; *η^2^* = 0.047), whereas women with low levels of anxiety and avoidance had a significantly higher ‘need for changes’ following childbirth (*F* = 5.421; *p* = 0.022; *η^2^* = 0.063). Statistical analysis showed a higher level of prenatal typicality than postnatal typicality in women with high levels of anxiety and avoidance (*F* = 4.277; *p* = 0.042; *η^2^* = 0.050) and in women with low anxiety and high avoidance (*F* = 6.508; *p* = 0.013; *η^2^* = 0.074). Women with high levels of anxiety and low avoidance were characterized by higher levels of ‘need for accomplishments’ (*F* = 4.548; *p* = 0.036; *η^2^* = 0.053), ‘need for affiliation’ (*F* = 4.684; *p* = 0.033; *η^2^* = 0.055), and ‘critical parent’ (*F* = 4.355; *p* = 0.040; *η^2^* = 0.051) during pregnancy. In women with a safe attachment style (low anxiety, low avoidance), a significant decrease in the level of variable ‘leadership’ postpartum was observed (*F* = 4.496; *p* = 0.037; *η^2^* = 0.053).

Question 2: In women with different maternal attachment styles, does the image of one’s mother as a mother change following childbirth?

In order to answer this research question, we used a statistical analysis analogous to that used in the first question. The dependent variable was the image of participants’ mother as a mother.

The results of the analysis showed that following childbirth, the participants’ image of their mother as a mother changed on the following subscales: ‘need for accomplishments’ (*F* = 4.655; *p* = 0.034; *η^2^* = 0.054), ‘need for domination’ (*F* = 10.033; *p* = 0.002; *η^2^* = 0.111), and ‘self-confidence’ (*F* = 5.234; *p* = 0.025; *η^2^* = 0.061). For each of these variables, the results were higher before childbirth.

For most subscales, the effect of the ‘attachment style’ between-subjects variable turned out to be significant. Simple contrast analysis was performed, where the reference group was women with a safe attachment style (low anxiety, low avoidance). Statistically significant differences were noted on the following subscales: ‘positive adjectives’, ‘negative adjectives’, ‘typicality’ ‘need for perseverance’, ‘need for understanding oneself and others’, ‘need for caring’, ‘need for affiliation’, ‘need for heterosexual contacts’, ‘need for aggression’, ‘self-confidence’, ‘personal adaptation’, ‘perfect me’, ‘leadership’, ‘femininity’, ‘caring parent’, ‘adult’, and ‘subordinate child’ (see Table 2). In all of these subscales, the observed differences related to the image of one’s mother as a mother in women with a safe attachment style and in women with high levels of anxiety and avoidance. Additionally, the image of one’s mother as a mother differentiated between women with low levels of anxiety and high levels of avoidance from women with safe attachment style on the following subscales: ‘positive adjectives’, ‘need for perseverance’, ‘need for understanding oneself and others’, ‘need for caring’, ‘self-confidence’, ‘perfect me’, ‘leadership’, ‘caring parent’, ‘adult’, and ‘submissive child’. It can be concluded, therefore, that the image of one’s mother as a mother in the examined women was dependent on the level of avoidance.

A statistically significant effect of interaction was obtained in relation to the ‘personal adjustment’ variable (*F* = 3.185; *p* = 0.028; *η^2^* = 0.106). Women with a safe attachment style achieved a significantly lower result after childbirth (*F* = 14.282; *p* < 0.001; *η^2^* = 0.150). They also had a higher level of the ‘image of one’s mother as a mother’ in relation to the tested variable than the participants with a high level of anxiety and a high level of avoidance (*F* = 5.881; *p* = 0.001; *η^2^* = 0.179).

The analysis of simple effects showed that the image of one’s mother as a mother changed after the participants gave birth. Women with a safe attachment style achieved higher scores in the first stage of the study: ‘complete list of adjectives’, ‘positive adjectives’, ‘typicality’, ‘need for accomplishments’, ‘need for perseverance’, ‘need for order’, ‘need for understanding oneself and others’, ‘need for affiliation’, ‘personal adaptation’, ‘femininity, ‘adult’ and ‘low originality-high intelligence’ (see Table 3). For these participants, the level of the variable ‘submissive child’ increased after childbirth (see Table 3).

Women with high level of anxiety and low avoidance achieved higher results in the subscale ‘need for heterosexual contacts’ (*F* = 4.258; *p* = 0.042; *η^2^* = 0.050). In women with low levels of anxiety and high levels of avoidance after childbirth, the variable scores decreased for the subscales: ‘complete list of adjectives’, ‘need for accomplishments’, ‘need for domination’, ‘need for heterosexual contacts’, ‘femininity’, ‘critical parent’, ‘high originality-low intelligence’, ‘high originality-high intelligence’, ‘low originality-low intelligence’, and ‘low originality-high intelligence’ (see Table 4).

Question 3: In women with different maternal attachment styles, does the bond with the child change following childbirth?

In order to answer this research question, we conducted a hierarchical regression analysis. This type of analysis takes into account the fact that the measurement of the bond with the child before and after childbirth was carried out using two different tools. The dependent variable in the tested model was the bond with the newborn child (measured by the PBQ test). The predictor introduced in the first block was the connection with the child during pregnancy (measured by the MFAS test), and the predictors introduced in the second block were: anxiety-ambivalent style and avoidance style. The obtained results indicated that the bond with the child during pregnancy was a significant predictor of the bond with the child after birth (see Table 5). The level of anxiety-ambivalent style and avoidance style were not important predictors for the mother’s bond with the newborn child (see Table 5). Although the model including three tested predictors turned out to be statistically significant, the introduction of two insignificant predictors weakened the significance of the model with one predictor (see Table 5).

## 4. Discussion

The results presented here allow us to explore the dynamics of the relationship between one’s self-image as a mother, the image of one’s mother as a mother, and the bond with a child during pregnancy and following childbirth.

The verification of Question 1 (In women with different maternal attachment styles, does self-image as a mother change following childbirth?) indicates that, before childbirth, women—in contrast to women after childbirth, regardless of attachment style—presented themselves, on the one hand, as more critical towards themselves and others, and on the other hand, as more conscientious, caring for the common good, more relaxed in social situations, straightforward, and comfortable with others. They also attributed more positive attributes to themselves.

During pregnancy, especially during the first pregnancy, a woman receives a considerable amount of attention and gratification from those around her. Future mothers might have many fantasies about their future and about their role as a mother, but these are their main fantasies [8]. The study was mainly carried out in antenatal clinics, so it can be concluded that the participants actively sought information and were open to learning about themselves and their relationships [30].

The reality of a mother with a small child is quite different. A mother’s assessment of herself and her needs can be changed by fatigue, the pressure resulting from the child’s needs, and frustration resulting from her own needs. At this time, a shift in a mother’s thinking about herself occurs—from ‘me as a pregnant woman’ to the lasting ‘me as a mother’ [31]. It is also worth noting that our results seem to be consistent with the idea that during pregnancy women seek information, both about their pregnancy and themselves, in order to build their maternal identity, but after childbirth they get information directly from their relationship and interaction with the child [32]. Following childbirth, a woman focuses less on herself and more on her relationship with the child. In this context, the general tendency of women after childbirth to choose a smaller number of adjectives is understandable.

The variability of the ‘need for change’ depending on the measurement and style of attachment is an interesting result. In women with a safe maternal attachment style, this need increases after childbirth; in women with high levels of anxiety and avoidance, it decreases. This is a puzzling result, especially if one considers the better adaptation of women with a safe style of maternal attachment to motherhood, and the ability to benefit from the support of an internal and real aspects of a relationship with their mother [10]. Perhaps this is due to the greater access of women with a safe style of attachment to the difficult aspects of motherhood, their frustration and fatigue, which can manifest themselves in a desire to change their lives.

The verification of Question 2 (In women with different maternal attachment styles, does the image of one’s mother as a mother change following childbirth?) brought an interesting result concerning the perceptions of mothers after birth as being less dominant, less self-confident, and less achievement-oriented. This result can be understood in the sense that, following childbirth, women become, in a way, equal, to their own mothers. A woman, as well as being a child of her mother, becomes the mother of her own child and this affects her relationship with her own mother and thus the image of her mother as a mother—the relationship with the mother is thus redefined [33].

The most important result seems to be the one indicating that the image of one’s mother as a mother depends on the level of avoidance in attachment, regardless of the measurement. The more one avoids attachment, the more negative the image of one’s mother is. This is a very important result because, in the absence of an internal, positive image of the mother, there is nothing to draw on to build one’s own positive image as a mother [34]. Moreover, the negative image of the mother is linked to the bond with the child during pregnancy [35], the bond with the child following childbirth [10], and the experience of motherhood as highly stressful [36]. Moreover, the relationship with one’s own mother seems to play an important role in the formation and course of postnatal depression [23]. A safe maternal attachment style and perceiving one’s mother as caring is believed to be a protective factor [21].

The verification of Question 3 (In women with different maternal attachment styles, does the bond with the child change following childbirth?) showed that the main predictor of the bond with the child following childbirth was the bond with the child during pregnancy. Problems with the bond following childbirth negatively correlated with the taking on of a parental role, treating the child as a separate entity, establishing interaction with the child, assigning properties to the child, and with the bond with the child during pregnancy. What the mother thinks about her baby during pregnancy is important to her way of building a relationship with the baby following childbirth. Rossen et al. [37] obtained similar results: bonding increased significantly in quality and intensity throughout the pregnancy. Stronger antenatal bonding at all time points (trimesters one through three) predicted stronger postnatal bonding.

These data underline the importance of preventive action and therapeutic efforts during pregnancy. It is worth noting that, in Poland, screening tests concerning the risk and intensification of the symptoms of depression during pregnancy have only been carried out since 1 January 2019 [38]. The Polish Ministry of Health is planning to introduce training programs and supervision schemes for midwives in response to this change and the resultant new responsibilities.

## 5. Strength and Limitations of this Study

The biggest strength of this study is its ability to trace the relationship between the studied variables over time. It is also important to take into account many aspects of the studied process, including that the impact of perinatal depression on the measurement was controlled.

The number of participants, especially the number of women who dropped out of the study, could be a significant limitation. It is also worth adding that it may not be possible to generalize our conclusions onto a wider population because most of the participants where highly educated women living in a big city. The influence of other people on the transition to motherhood was not controlled in our study. It is worth adding that the study took place in Poland and all of the participants were Polish, which may also limit the ability to generalize the results as culture plays an important role in issues connected to attachment patterns [39,40,41,42].

## 6. Conclusions

Our study indicates that the relationships of different maternal attachment styles, self-image as a mother, and the image of one’s mother as a mother during and after pregnancy influence the processes of bonding with the child following childbirth. The presented results illustrate the dynamics of one’s self-image as a mother, the image of one’s mother as a mother, and the bond with the child depending on one’s maternal attachment style. Despite the small size of the sample, it can be concluded that these variables are in strong interaction with one other. It is important that those who support pregnant women and new mothers are aware of these complex processes of becoming a mother and, if necessary, provide suitable support and refer women to specialists (e.g., support groups for new mothers, trained midwives, psychologists, psychotherapists, psychiatrists).

Particular attention should be paid to women’s narratives regarding their relationships with loved ones; in particular, young women with high avoidance—those with a negative image of their mothers—may experience greater difficulties during this period due to strategies which involve deactivation of the need for intimacy.

## Figures and Tables

**Table 1:** Significant effects of the main factor ‘measurement’ on self-image as a mother (mixed Analysis of Covariance (ANCOVA)).

Variable	*F*	*p*	*η^2^*
Complete list of adjectives	6.602	0.012	0.075
Negative adjectives	7.349	0.008	0.083
Typicality	6.848	0.011	0.078
The need for affiliation	4.070	0.047	0.048
Leadership	5.296	0.024	0.061
Critical parent	8.892	0.004	0.099

**Table 2:** Significant effects of the main factor ‘attachment style’ on the image of one’s mother as a mother (mixed ANCOVA).

Variable	*F*	*p*	*η^2^*
Positive adjectives	5.329	0.002	0.165
Negative adjectives	5.490	0.002	0.169
Typicality	6.052	0.001	0.183
Need for perseverance	3.734	0.014	0.122
Need for understanding oneself and others	3.677	0.015	0.120
Need for caring	7.633	0.001	0.220
Need for affiliation	5.320	0.002	0.165
Need for heterosexual contacts	6.322	0.001	0.190
Need for aggression	2.958	0.037	0.099
Self-confidence	4.531	0.005	0.144
Personal adaptation	5.881	0.001	0.179
Perfect me	6.269	0.001	0.188
Leadership	6.092	0.001	0.184
Femininity	5.289	0.002	0.164
Caring parent	7.866	0.001	0.226
Adult	3.656	0.016	0.119
Subordinate child	5.026	0.003	0.157

**Table 3:** Significant simple effects among women with safe attachment style on image of one’s mother as a mother (mixed ANCOVA).

Variable	*F*	*p*	*η^2^*
Complete list of adjectives	7.183	0.009	0.081
Positive adjectives	10.489	0.002	0.115
Typicality	4.229	0.043	0.050
Need for accomplishments	6.994	0.010	0.079
Need for perseverance	5.322	0.024	0.062
Need for order	11.097	0.001	0.120
Need for understanding oneself and others	9.256	0.003	0.103
Need for affiliation	9.324	0.003	0.103
Personal adaptation	14.282	0.001	0.150
Femininity	4.501	0.037	0.053
Adult	9.995	0.002	0.110
Low originality-high intelligence	10.512	0.002	0.115
Submissive child	5.193	0.025	0.060

**Table 4:** Significant simple effects among women with low levels of anxiety and high levels of avoidance on image of one’s mother as a mother (mixed ANCOVA).

Variable	*F*	*P*	*η^2^*
Complete list of adjectives	7.723	0.007	0.087
Need for accomplishments	5.398	0.023	0.062
Need for domination	4.212	0.043	0.049
Need for heterosexual contacts	5.700	0.019	0.066
Femininity	5.206	0.025	0.060
Critical parent	4.519	0.037	0.053
High originality-low intelligence	4.595	0.035	0.054
High originality-high intelligence	8.477	0.005	0.095
Low originality-low intelligence	5.387	0.023	0.062
Low originality-high intelligence	4.027	0.048	0.047

**Table 5:** Hierarchical regression analysis with the ‘bond with the newborn child’ (measured by the Postpartum Bonding Questionnaire (PBQ) test) as a dependent variable.

	Variable	β	*t*	*p*	*R^2^*	*F*	*p*
First block	Bond with the child during pregnancy	−0.289	2.72	0.008	0.085	7.77	0.007
Second block	Anxiety-ambivalent style	0.192	1.80	0.076	0.123	3.85	0.012
	Avoidance style	0.016	0.146	0.884			
